# NUMB and NUMBL differences in gene regulation

**DOI:** 10.18632/oncotarget.24186

**Published:** 2018-01-11

**Authors:** José Manuel García-Heredia, Amancio Carnero

**Affiliations:** ^1^ Instituto de Biomedicina de Sevilla, IBIS, Hospital Universitario Virgen del Rocío, Universidad de Sevilla, Consejo Superior de Investigaciones Científicas, Seville, Spain; ^2^ Department of Vegetal Biochemistry and Molecular Biology, University of Seville, Seville, Spain; ^3^ CIBER de Cáncer, Instituto de Salud Carlos III, Pabellón 11, Planta 0, Madrid, Spain

**Keywords:** NUMB, NUMBL, cancer, Notch pathway, WNT pathway

## Abstract

NUMB, and its close homologue NUMBL, behave as tumor suppressor genes by regulating the Notch pathway. The downregulation of these genes in tumors is common, allowing aberrant Notch pathway activation and tumor progression. However, some known differences between NUMB and NUMBL have raised unanswered questions regarding the redundancy and/or combined regulation of the Notch pathway by these genes during the tumorigenic process. We have found that NUMB and NUMBL exhibit mutual exclusivity in human tumors, suggesting that the associated tumor suppressor role is regulated by only one of the two proteins in a specific cell, avoiding duplicate signaling and simplifying the regulatory network. We have also found differences in gene expression due to *NUMB* or *NUMBL* downregulation. These differences in gene regulation extend to pathways, such as WNT or Hedgehog. In addition to these differences, the downregulation of either gene triggers a cancer stem cell-like related phenotype. These results show the importance of both genes as an intersection with different effects over cancer stem cell signaling pathways.

## INTRODUCTION

NUMB and NUMBL, or NUMB-Like, belongs to a closely conserved family of proteins with important roles in a large variety of cellular processes ranging from cell adhesion to ubiquitination [1–7]. *NUMB*, the first gene in the family identified in *Drosophila* [8], has been suggested to play a role in asymmetric division, allowing cell differentiation [7]. Most research conducted to date has been focused on the role of NUMB, assuming that NUMBL performs the same functions, although NUMBL does not display an asymmetric distribution in cells during mitosis [9]. In addition, its expression is different during development, with ubiquitous NUMB expression and more restricted expression of NUMBL in the central nervous system [6, 9–11]. Knock-out experiments in mice have shown that, although *NUMBL* deletion showed no differences during embryogenesis, the deletion of *NUMB* or combined *NUMB*/*NUMBL* deletion were embryonic lethal [6, 12, 13]. Together, these differences show that, although *NUMB* and *NUMBL* have a conserved structure and domains [14], the functional differences between the proteins must also be considered.

*NUMB* and *NUMBL* have been characterized as tumor suppressor genes [15–17], leading to Notch signaling pathway inhibition [4, 17] or p53 stabilization [18, 19]. NUMB inhibits the Notch pathway through its interaction with ITCH and NICD (Notch IntraCellular Domain), labeling NICD for ubiquitination and degradation [4, 20–22]. Although this is one of the most known roles of NUMB, this protein has also been linked to the WNT pathway, promoting β-catenin degradation through polyubiquitination [23]. The role of NUMB as a tumor suppressor gene has been widely characterized, revealing that lower NUMB levels are associated with a worse prognosis in malignant pleural mesothelioma [24]. In addition, different tumors, such as breast cancer, salivary gland carcinoma, non-small-cell lung carcinoma or medulloblastoma, also exhibit a downregulation of NUMB expression [25–28]. Conversely, NUMB overexpression reduces cell proliferation and increases cell sensitivity to cisplatin [24, 25, 29].

Previous results obtained by *NUMBL* knockdown by shRNA, with no changes in *NUMB* levels, showed an increment in tumorigenic properties and increased resistance to chemotherapy, with a worse prognosis in breast, lung and colorectal tumors [17]. Importantly, the downregulation of *NUMBL* also triggers Notch pathway activation, further increasing the epithelia-mesenchymal transition (EMT), cancer stem cell (CSC) transcriptional markers and CSC-like phenotypes. *NUMBL* has also been described as a tumor suppressor gene, mainly based on its ability to inhibit the Notch pathway [17, 30, 31]. However, NUMBL can also activate Hedgehog signaling, which represents a functional difference compared with NUMB [32]. According to these results, NUMBL can activate Hedgehog signaling and thus increase the stem cell population. This phenomenon suggests that, under certain circumstances, NUMBL could act as an oncogene. This process has also recently been described for NUMB, showing that an altered isoform expression is common in cancer cells [23, 33–35].

A small percentage of human tumors exhibit lower NUMBL expression than normal tissue, being this reduced expression associated with a poor prognosis and worse patient survival [30, 31]. Inhibition of only one NUMB family protein is sufficient to modify cancer cell properties, since a partial decrease in NUMB or NUMBL is sufficient to increase Notch pathway activation and cancer stem-like properties. This phenomenon suggests that NUMB and NUMBL act as essential regulators of cancer cell properties, individually acting in a dose-dependent manner and regulating the same pathway with a certain degree of redundancy. Like NUMB, NUMBL seems to regulate Notch pathway activity [36, 37]. It is interesting to note that the downregulation of only one of these proteins, either NUMB or NUMBL, is sufficient to allow Notch pathway activation, increasing the pool of CSC-like cells [38–41]. However, whether there is a dose effect of combined NUMB/NUMBL or whether the downregulation of both proteins may coexist in some very aggressive tumors remains unknown. Such an additive effect still must be demonstrated, as well as whether the accumulative effects are linear or reach a certain threshold with no further increases in tumorigenicity. Finally, the effects of the distinct tumor suppressor activities on different pathways in cells must be elucidated.

Here, we showed the mutual exclusivity of NUMB and NUMBL in tumors and the opposite gene regulation affecting at least three different signaling pathways: Notch, WNT and Hedgehog. Although both genes equally regulate Notch, the WNT and Hedgehog pathways are oppositely regulated by NUMB and NUMBL, according to our gene transcription analysis. However, the final phenotypic endpoint induced by *NUMB* or *NUMBL* downregulation was similar, suggesting some hierarchy in the signaling pathways.

## RESULTS

### *NUMB* and *NUMBL* show a negative correlation in tumors

**Figure 1:**
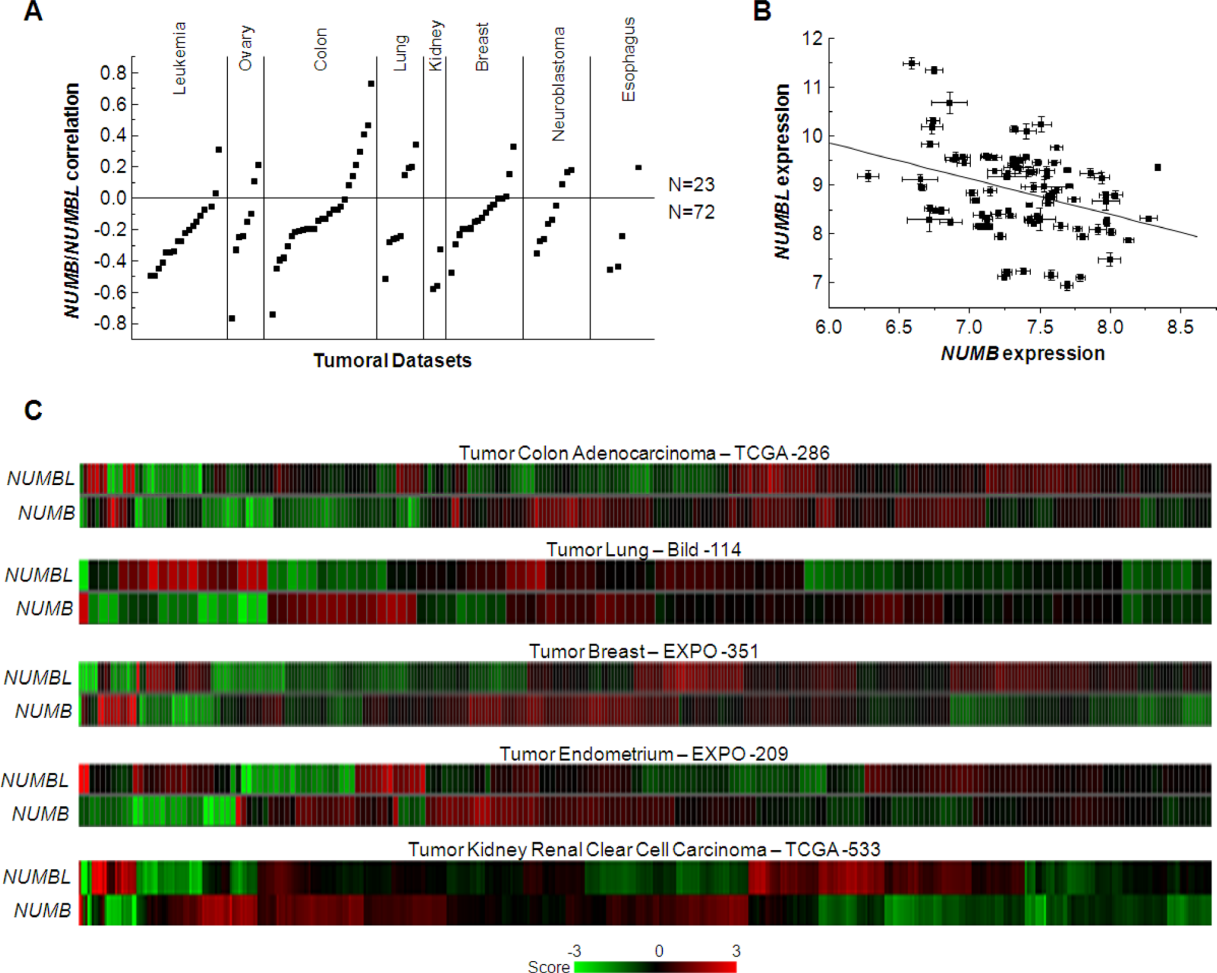
*NUMB* and *NUMBL* are negatively correlated in human tumors (**A**) *NUMB*/*NUMBL* correlations in leukemia, ovarian, colon, lung, kidney, breast, neuroblastoma and esophagus tumors, showing a higher percentage of negative correlations. (**B**) Correlation of *NUMB* and *NUMBL* expression in human tumors, showing a significant Pearson correlation coefficient (*R* = −0.355, *p* = 4.4 × 10^−4^). (**C**) Heat map of *NUMB* and *NUMBL* expression in human tumors.

**Figure 2:**
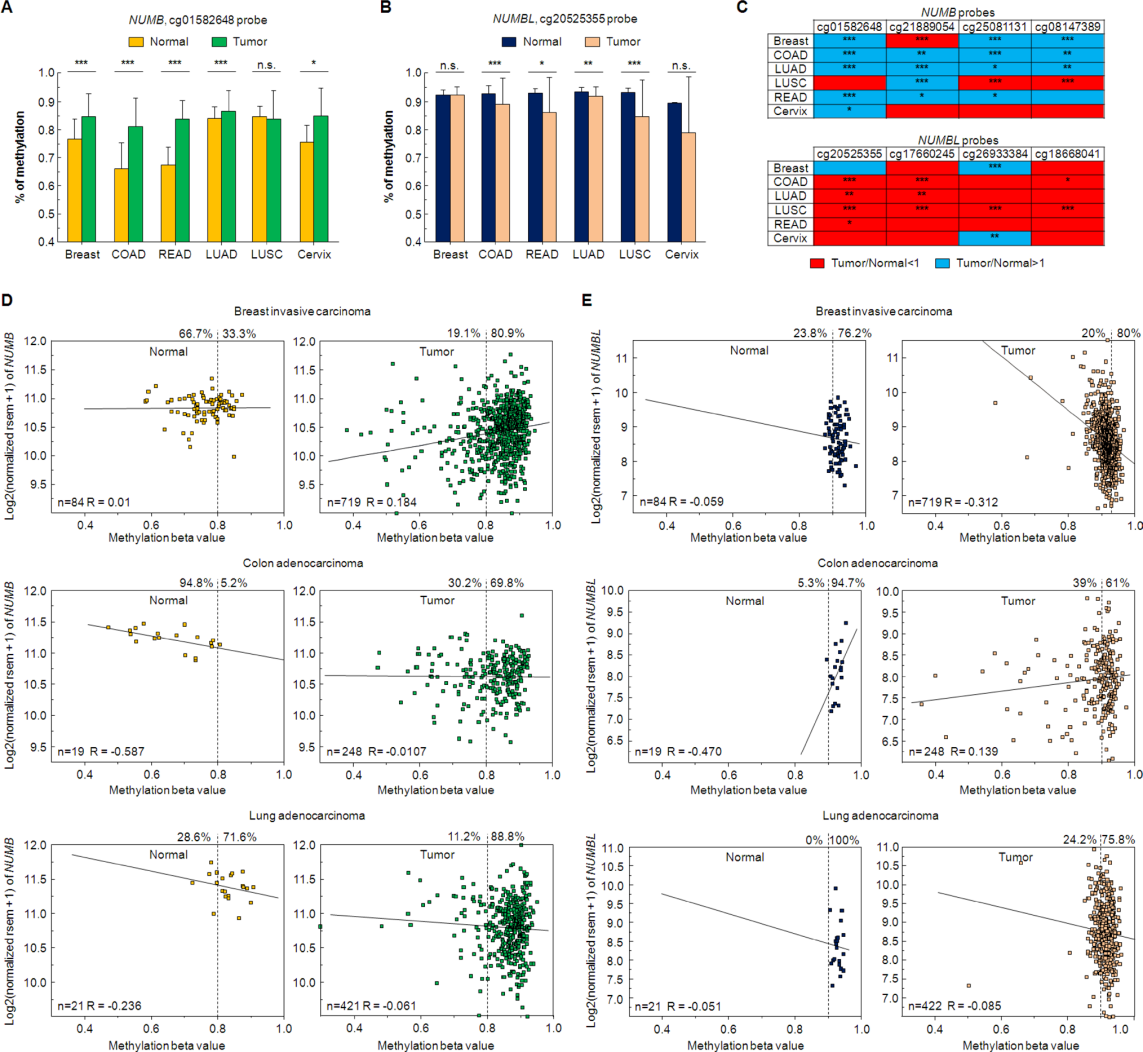
Changes in NUMB/NUMBL methylation in tumoral samples (**A**) Percentage of *NUMB* methylation for cg0158648 probe in six different tumors. Breast: Breast invasive carcinoma; COAD: Colon adenocarcinoma; READ: Rectum adenocarcinoma; LUAD: Lung adenocarcinoma; LUSC: Lung squamous cell carcinoma; Cervix: cervical squamous cell carcinoma and endocervical adenocarcinoma. (**B**) Percentage of *NUMBL* methylation for cg20525355 probe in six different tumors. (**C**) *NUMB* and *NUMBL* are differentially methylated in normal or tumoral samples. Red color: tumoral samples with a lower methylation value regarding normal samples. Blue color: tumoral samples with a higher methylation value regarding normal samples. (**D**) Relation between expression and methylation value for *NUMB*, using cg0158648 probe, in breast invasive carcinoma, colon adenocarcinoma and lung adenocarcinoma. (**E**) Relation between expression and methylation value for *NUMBL*, using cg20525355 probe, in breast invasive carcinoma, colon adenocarcinoma and lung adenocarcinoma. The Student’s *T* test was used to determine significant differences (^*^= *p <* 0.05; ^**^= *p* < 0.01; ^***^= *p* < 0.001).

As mentioned above, NUMB and NUMBL are commonly described as proteins with a very similar function, characterized as tumor suppressors. To identify any differential effects in tumors, we analyzed a total of 95 datasets in ten different tumors (Acute Lymphoblastic Leukemia, Acute Myeloid Leukemia, Chronic Lymphocytic Leukemia, ovarian colon/rectal, lung, renal breast, neuroblastoma or esophageal tumors) using R2 software. We observed a clearly marked trend between *NUMB* and *NUMBL* since were negatively correlated in 72 of the 95 considered datasets (Figure 1A). Indeed, the correlation of *NUMB* and *NUMBL* expression provided a significant Pearson value (*R* = −0.355, *p* = 4.4 × 10^−4^) (Figure 1B), as clearly shown in the heat maps of tumors from colon, lung, breast, endometrium or kidney, in which individual samples can be compared (Figure 1C). These results, which were obtained directly from human tumor samples, showed that when one of these genes was highly expressed in tumors, the other was expressed at a lower rate. In fact, we also explored datasets derived from normal samples. We found that the negative correlation of *NUMB*/*NUMBL* was also present in non-tumoral samples, showing that both genes are to some extent mutually exclusive in cells (Supplementary Figure 1). This result suggests a stringent gene regulation to avoid undesirable effects. These differences might be due to differences in gene methylation, allowing higher expression of one of the genes in cells. To explore this phenomenon, we looked for changes in *NUMB* or *NUMBL* methylation using TCGA Wanderer resource [42]. By doing this, we found that *NUMB* is highly methylated in tumors regarding control (Figure 2A). However, the opposite happens with *NUMBL*, being highly methylated in normal samples but not in tumoral samples (Figure 2B). Differences in *NUMB* or *NUMBL* methylation between normal and tumoral samples appear to be general, as can be deduced from the fact that a common patron is obtained using four different CG probes (Figure 2C). At that way, for *NUMB*, methylation is higher in tumoral samples regarding control samples, while for *NUMBL* occurs the opposite. In addition, if we focus in the probe cg01582648, the percentage of tumoral samples with a methylation beta value higher than 0.8 is significantly higher regarding normal samples in breast invasive carcinoma (80.9% vs. 33.3%), colon adenocarcinoma (69.8% vs. 5.2%) and lung adenocarcinoma (88.8% vs. 71.6%) (Figure 2D). Again, the opposite happen for *NUMBL* probe cg20525355, being tumoral samples lower methylated regarding normal samples in colon adenocarcinoma (61% vs. 94.7%) and lung adenocarcinoma (75.8% vs. 100%) (Figure 2E). These results point to the possibility that *NUMB* or *NUMBL* might be preferentially expressed in a specific cell or tissue. Furthermore, suggest certain redundancy in the roles.

### *NUMB* and *NUMBL* have few common genes with the same correlations

**Figure 3:**
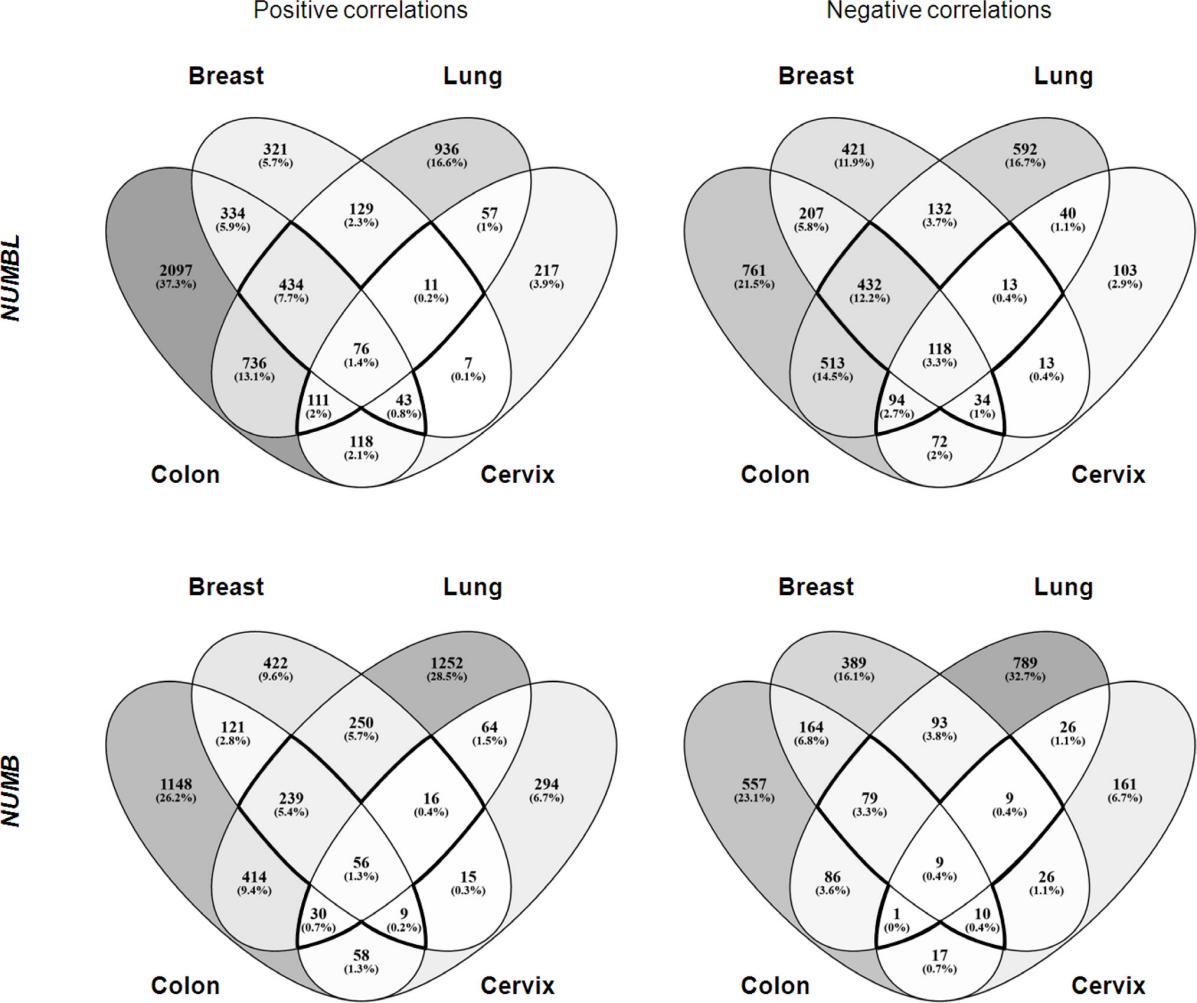
Venn diagram of genes positively and negatively correlated to *NUMB* or *NUMBL* in breast, lung, colon and cervix tumors (*p*-value < 0.05)

**Figure 4:**
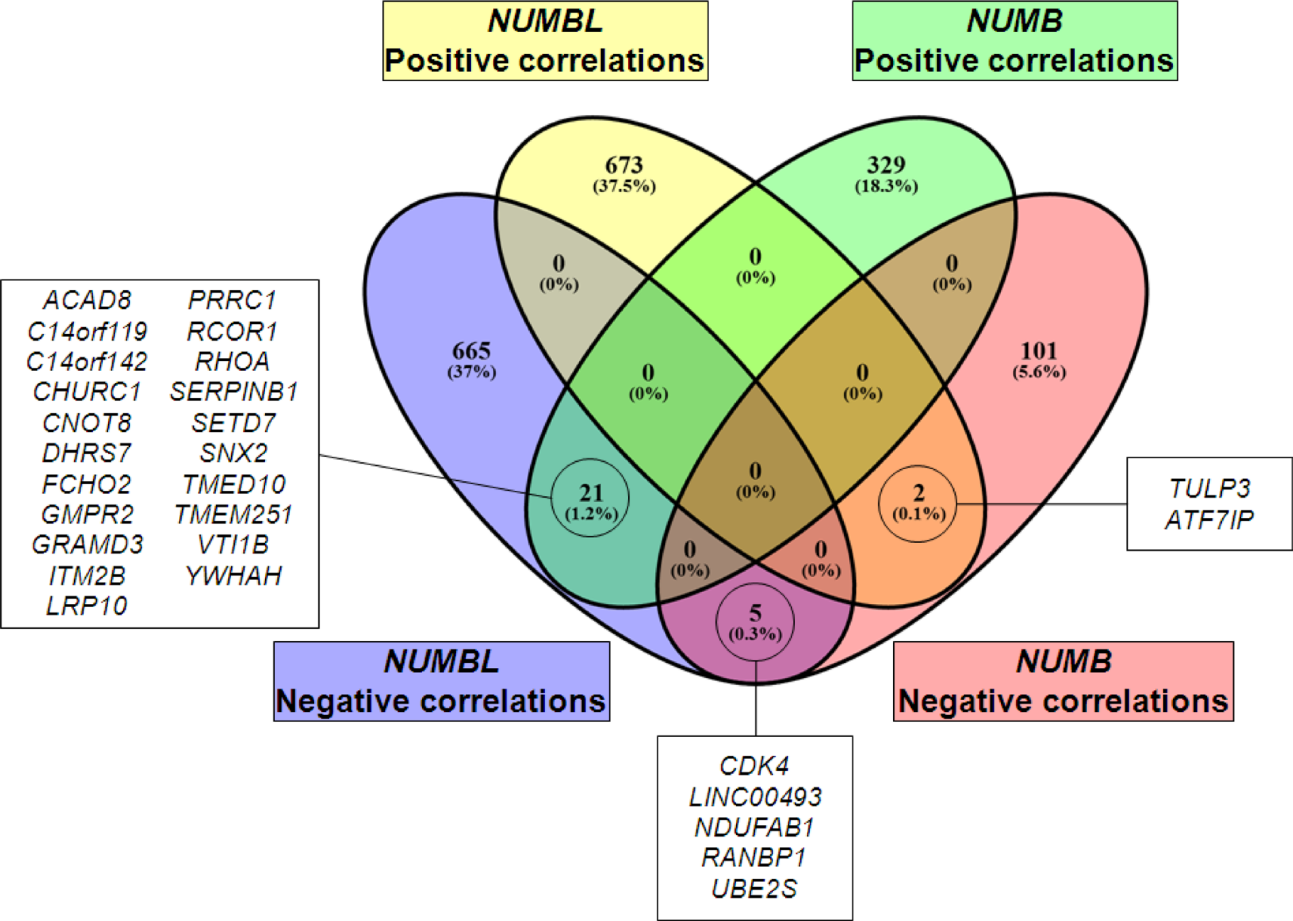
Venn diagram of genes positively or negatively correlated to *NUMB* or *NUMBL*, showing coincident genes

To identify the genes that correlated to *NUMB* and/or *NUMBL* in the same tumors, we performed the following analysis. We first selected public datasets from transcriptomic analyses of lung, cervical, breast and colorectal tumors (Supplementary Table 1). Next, we determined the genes that correlated to *NUMB* or *NUMBL* in all considered tumor types and then compared the individual tumor types to obtain a map of common genes correlating to *NUMB* or *NUMBL* expression in various tumors. We found 675 genes that were positively correlated and 691 genes that were negatively correlated to *NUMBL* in at least three of the considered tumor types. Regarding *NUMB*, we found 350 genes that were positively correlated and 108 genes that were negatively correlated, taking into account at least three of the tumors (Figure 3, Supplementary Table 2). Due to the previously assumed overlapping functions of NUMB and NUMBL in cells, it would be expected to find genes that were positively or negatively correlated to *NUMB* and *NUMBL*. In our bioinformatics analysis of tumors considering all tumor types, no common genes from public transcriptome tumor datasets were positively correlated both to *NUMB* and *NUMBL*. However, we obtained a short list of 5 genes that were negatively correlated both to *NUMB* and *NUMBL* (as expected from its behavior as tumor suppressors), a second list of 21 genes that were positively correlated to *NUMB* and negatively correlated to *NUMBL*, and two genes negatively correlated to *NUMB* and positively correlated to *NUMBL* (Figure 4). However, when we compared the genes that were correlated to *NUMB* or *NUMBL* in each individual tumor type, we found common positively or negatively correlated genes, suggesting that NUMB and NUMBL participate in different gene regulatory processes, depending on the tissue (Supplementary Table 3).

### Gene ontology analysis of NUMB and/or NUMBL correlations

Next, we performed Gene Ontology (GO)-term and Reactome enrichment analysis, considering separately the genes correlated to *NUMB* or *NUMBL*, due to the low number of common genes (Supplementary Table 4–7). Interestingly, we obtained common GO and Reactome terms for genes negatively correlated with *NUMB* or *NUMBL* (Supplementary Table 8). In particular, we found negative correlations with protein synthesis (from ribosome to mRNA maturation), general metabolism or protein transport. In addition, we found Reactome terms related to cell cycle, suggesting a possible deregulation due to changes in *NUMB* or *NUMBL* expression (Supplementary Table 8). At that way, one of the common genes negatively correlated both to *NUMB* and *NUMBL* was *CDK4*, a known gene connected to cell division. In addition, we also found components (*MCM3* for *NUMBL* and *MCM2* for *NUMB*) of the minichromosome maintenance protein (MCM) complex, necessary for cell cycle transition to S-phase [43]. These results can be related to the functions of *NUMB* and *NUMBL* as negative regulators of cell division, supporting their roles as tumor suppressor genes.

From the list of genes that positively correlated with either *NUMB* or *NUMBL*, similar GO-terms could not be obtained. However, it is interesting to note that, regarding *NUMB*, some GO-terms were related to vesicle transport, a previously known *NUMB* function [44, 45] (Table 1). However, *NUMBL* appeared to be positively correlated to genes related to gene regulation and RNA metabolism (Table 2), suggesting an unexplored role for this gene.

**Table 1:** GO-terms obtained from genes positively correlated with *NUMB*

GO-term	Fold enrichment	*p*-value
vesicle targeting	8.47	2.76 × 10^−4^
COPII vesicle coating	8.37	0.0161
vesicle targeting, rough ER to cis-Golgi	8.37	0.0161
vesicle coating	8.12	0.0207
COPII-coated vesicle budding	7.87	0.0264
vesicle targeting, to, from or within Golgi	7.76	0.0298
actomyosin	6.9	0.0372
establishment of vesicle localization	4.76	3.19 × 10^−4^
vesicle localization	4.53	6.87 × 10^−4^
Golgi vesicle transport	4.04	2.34 × 10^−4^
Vesicle organization	3.47	0.0341
vesicle-mediated transport	2.27	5.75 × 10^−3^
Golgi subcompartment	2.27	0.0332
Golgi apparatus	2.08	5.66 × 10^−4^
cytoplasmic vesicle	1.85	4.58 × 10^−4^
intracellular vesicle	1.85	4.82 × 10^−4^
vesicle	1.53	1.58 × 10^−3^

**Table 2:** GO-terms obtained from genes positively correlated with *NUMBL*

GO-term	Fold Enrichment	*p*-value
transcription, DNA-templated	1.89	1.31 × 10^−8^
nucleic acid-templated transcription	1.89	1.35 × 10^−8^
RNA biosynthetic process	1.88	1.71 × 10^−8^
nucleobase-containing compound biosynthetic process	1.72	1.94 × 10^−6^
DNA binding	1.71	4.75 × 10^−5^
RNA metabolic process	1.64	3.86 × 10^−6^
regulation of RNA metabolic process	1.53	1.30 × 10^−4^
regulation of RNA biosynthetic process	1.51	1.25 × 10^−3^
regulation of transcription, DNA-templated	1.51	1.25 × 10^−3^

Genes that were negatively correlated to *NUMBL* resulted in a very extensive list of GO and Reactome terms, some of which were related to three different signaling pathways: NF-κB, WNT and Hedgehog, suggesting that, as previously reported [32], *NUMBL* could modulate the Hedgehog pathway (Table 3). We also found that *NUMBL* was negatively correlated to NF-kB signaling, mitochondrial tricarboxylic acid cycle (TCA) and p53 stabilization, as also previously described [30, 46, 47].

**Table 3:** GO and Reactome pathways terms related to Hedgehog, WNT and NF-κB pathways, obtained from genes negatively correlated with *NUMBL*

GO-term	Fold enrichment	*p*-value
NIK/NF-kappaB signaling	5.15	3.62 × 10^−3^
respiratory electron transport chain	4.55	4.37 × 10^−5^
Wnt signaling pathway, planar cell polarity pathway	4.68	5.27 × 10^−3^
**Reactome pathways term**	**Entities *p*-Value**	**Entities FDR**
Hedgehog ‘off’ state	1.72 × 10^−3^	0.0137
GLI3 is processed to GLI3R by the proteasome	3.06 × 10^−6^	9.18 × 10^−5^
Degradation of GLI2 by the proteasome	3.06 × 10^−6^	9.18 × 10^−5^
Degradation of GLI1 by the proteasome	3.06 × 10^−6^	9.18 × 10^−5^
Hedgehog ligand biogenesis	1.74 × 10^−5^	2.44 × 10^−4^
Hh mutants that don’t undergo autocatalytic processing are degraded by ERAD	1.16 × 10^−5^	2.06 × 10^−4^
Hh mutants abrogate ligand secretion	2 × 10^−5^	2.65 × 10^−4^
Beta-catenin independent WNT signaling	2.63 × 10^−3^	0.021
Degradation of beta-catenin by the destruction complex	1.7 × 10^−5^	2.44 × 10^−4^
Degradation of AXIN	5.47 × 10^−6^	1.26 × 10^−4^
Autodegradation of Cdh1 by Cdh1:APC/C	3.7 × 10^−6^	9.62 × 10^−5^
Dectin-1 mediated noncanonical NF-kB signaling	2.72 × 10^−5^	3.26 × 10^−4^
Activation of NF-kappaB in B cells	6.81 × 10^−5^	6.81 × 10^−4^
TNFR2 non-canonical NF-kB pathway	2.4 × 10^−3^	0.0192
NIK––>noncanonical NF-kB signaling	1.16 × 10^−5^	2.06 × 10^−4^
Citric acid cycle (TCA cycle)	2.58 × 10^−4^	2.58 × 10^−3^
The citric acid (TCA) cycle and respiratory electron transport	5.75 × 10^−9^	9.03 × 10^−7^
Stabilization of p53	1.69 × 10^−6^	7.61 × 10^−5^
p53-Dependent G1 DNA Damage Response	1.26 × 10^−5^	2.06 × 10^−4^

### *NUMB* and *NUMBL* show opposite correlations in some signaling pathways

**Figure 5:**
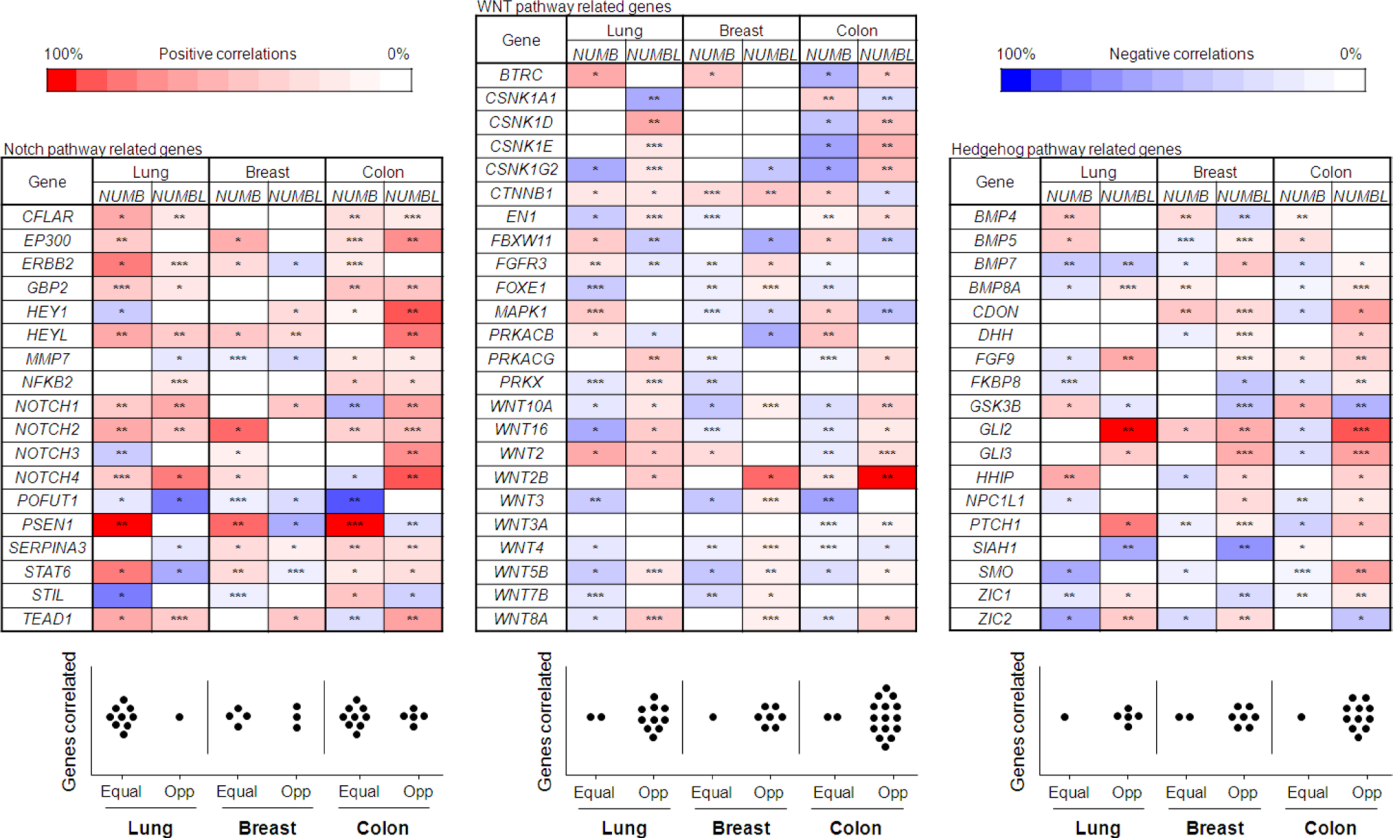
Upper figure: Notch, Hedgehog and WNT pathway genes with an opposite correlation for *NUMB* and *NUMBL* Statistical differences, obtained from gene correlations, are the followings: ^*^= *p* < 0.05; ^**^= *p* < 0.01; ^***^= *p <* 0.001. Bottom figure: Graph representative of the genes equally regulated vs. opposite regulated for each pathway in breast, lung and colon tumors.

NUMB and NUMBL have been previously described as inhibitors of the Notch pathway due to their interactions with NICD, allowing it to be labeled for ubiquitination and degradation. However, we did not find any GO or Reactome term clearly related to the Notch pathway, so we decided to search specifically for genes related to this pathway. We also searched for genes in the WNT and Hedgehog pathways since both pathways were negatively correlated with *NUMBL* (Table 3). A significant percentage of genes related to the Notch pathway showed the same behavior according to the presence of *NUMB* or *NUMBL* (Figure 5). Genes such as *CFLAR*, *EP300*, *GBP2*, *HEYL*, *KLF7*, *NOTCH1* or *POFUT* showed a similar correlation with *NUMB* or *NUMBL* in the three tumor types considered (Figure 5). However, many of the genes correlating with *NUMBL* showed an opposite behavior with *NUMB* in the other two analyzed pathways, WNT and Hedgehog (Figure 5), independently of the type of tumor. These results indicated that *NUMB* and *NUMBL* could differentially modulate different signaling pathways in a very different fashion. These results also indicate that besides the previously observed exclusivity, the full redundancy does not exist between these two genes. Only in the Notch pathway, equal regulation is maintained.

**Figure 6:**
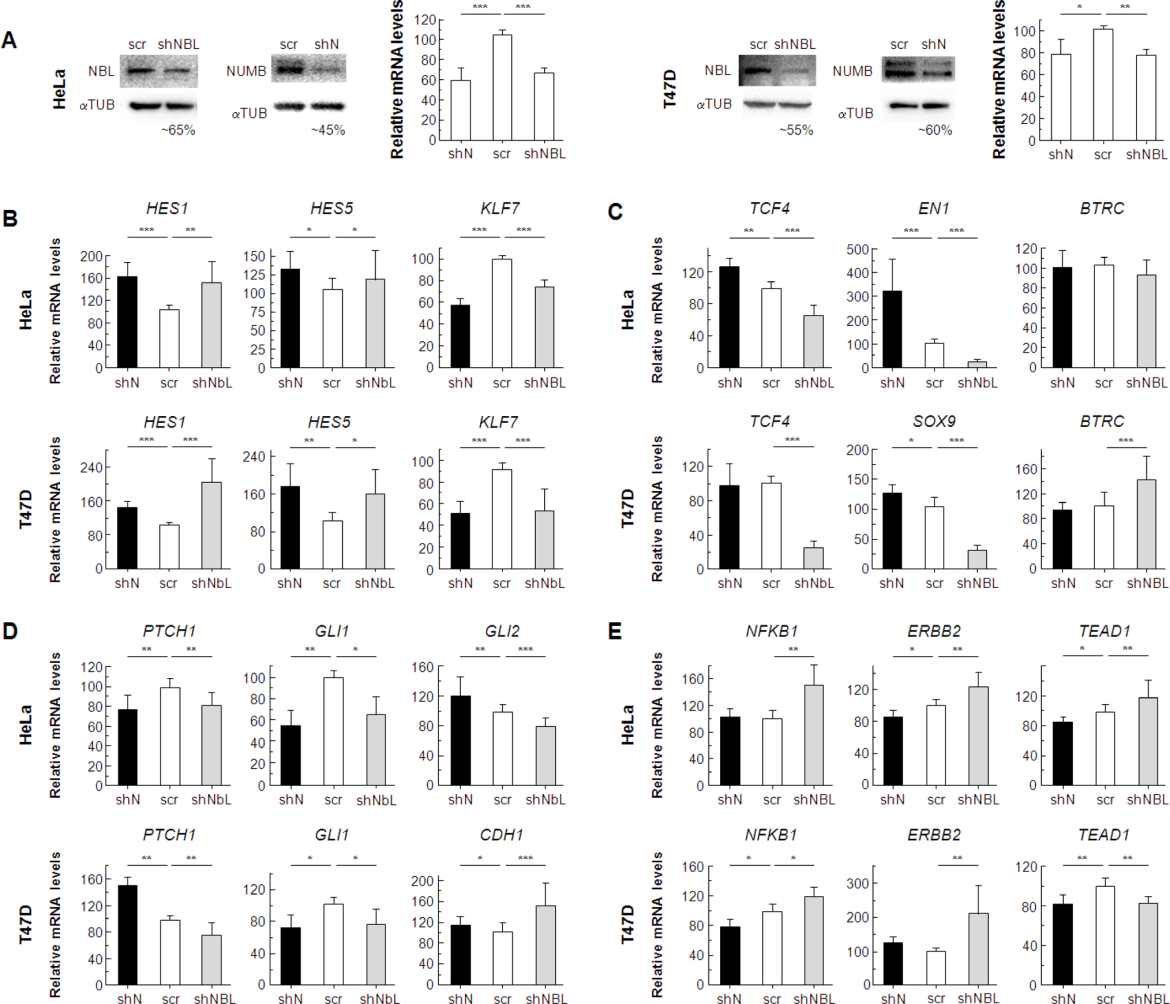
(**A**) Transfection of HeLa or T47D cells with *NUMB* or *NUMBL*-shRNA plasmids induces a decrease in protein and mRNA expression, as detected by WB and qPCR. (**B**) *NUMB* and *NUMBL* downregulation modifies mRNA levels of Notch pathway-related genes. (**C**) *NUMB* and *NUMBL* downregulation modify the expression of genes in the WNT pathway in an opposite manner. (**D**) Changes in Hedgehog related genes due to *NUMB* or *NUMBL* downregulation. (**E**) *NUMB* or *NUMBL* downregulation results in opposite changes in gene expression of the NFκB, ERBB/HER and Hippo pathways. All experiments were repeated a minimum of three independent times. The Student’s *T* test was used to determine significant differences (^*^= *p <* 0.05; ^**^= *p <* 0.01; ^***^= *p <* 0.001).

To experimentally assess this opposite gene regulatory role, we decided to analyze gene expression using T47D and HeLa cells transfected with specific shRNAs against *NUMB* or *NUMBL* (Figure 6A). Focusing on genes in the Notch pathway, we observed an equivalent activation of this pathway due to the downregulation of *NUMB* or *NUMBL* (Figure 6B), correlating with previous reports [17, 48]. Due to the relevance of the different gene associations with *NUMB* and *NUMBL*, we extended our analysis to the other two signaling pathways, WNT and Hedgehog. As expected from the bioinformatics analysis, we found opposite results due to the downregulation of *NUMB* or *NUMBL* in the mRNA levels of the target genes *TCF4*, *EN1*, *SOX9* and *BTRC*, which are considered read-outs of the WNT pathway (Figure 6C); or *PTCH1*, *GLI1*, *GLI2* and *CDH1*, considered read-outs of the Hedgehog pathway (Figure 6D).

Our bioinformatics results also led us to analyze other genes with opposite correlations in tumors, such as *NFκB1*, *ERBB2* and *TEAD1*. Measurement of the transcriptional responses of these genes to the reduced expression of *NUMB* or *NUMBL* revealed an opposite regulation of these genes in response to NUMB or NUMBL (Figure 6E), as expected based on our bioinformatics analysis. It is interesting to note that these three genes are part of other signaling pathways (NFκB, ERBB/HER and Hippo) [49–51], likely suggesting complex interaction of NUMB/L proteins with the signaling network.

### *NUMB* and *NUMBL* downregulation equally increases stem cell properties

**Figure 7:**
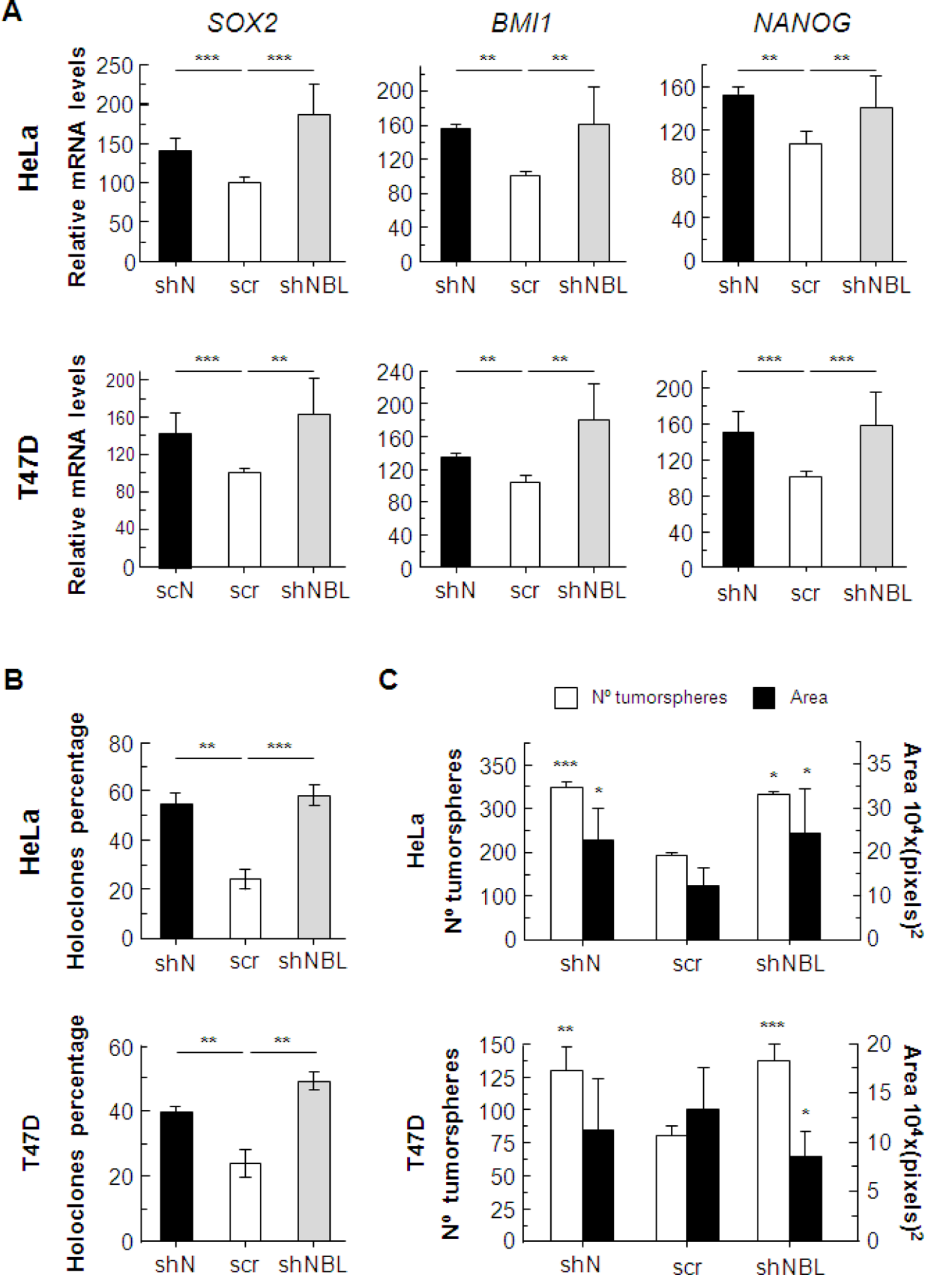
(**A**) Changes in the expression of genes related to stem cell properties due to *NUMB* or *NUMBL* downregulation. (**B**) Holoclone percentages are increased as a consequence of *NUMB* or *NUMBL* downregulation. (**C**) The number and area of tumorspheres are modified due to *NUMB* or *NUMBL* downregulation. All experiments were repeated a minimum of three independent times. The Student’s *T* test was used to determine significant differences (^*^= *p* < 0.05; ^**^= *p <* 0.01; ^***^= *p <* 0.001).

Although *NUMB* and *NUMBL* downregulation act differently regarding some of the considered genes, suggesting that *NUMB* and *NUMBL* target some different pathways, the global effect caused by their downregulation is very similar. To explore this point, we decided to measure the expression of some genes associated with the acquisition of stem cell-like properties and observed a clear increment in *SOX2*, *BMI1* and *NANOG* genes in response to both *NUMB* and *NUMBL* downregulation (Figure 7A), which have been previously related to stem cell properties [52–54]. The increment in the transcription of stem cell-like genes, together with the previously described increment in other stem cell-like genes related to *NUMB* or *NUMBL* downregulation, such as *OCT4* or *KLF4*, showed that, for all analyzed conditions, the cells acquire a more de-differentiated state, typical of stem-like cells [17, 48].

To rate these stem cell-like properties, we cultured cells at a low density, so that individual clones formed independent colonies. These clones can be classified as holoclones, meroclones and paraclones, according to their ability to reconstitute a tumor [55, 56]. Holoclones are considered to derive from cells with stem cell-like properties. We found that either *NUMB* or *NUMBL* downregulation caused similar increments in the percentages of holoclones of HeLa and T47D cells (Figure 7B). In addition, tumorspheres, colonies derived from stem cell-like cells that grow in suspension, showed a significant increment in number in both cell lines, demonstrating again that *NUMB*/*NUMBL* downregulation increased stem cell-like properties (Figure 7C), regardless of the differences in pathway activation.

## DISCUSSION

*NUMB* and *NUMBL* have been considered tumor suppressor genes with very similar functions because both can regulate the Notch pathway [5, 17, 22, 30, 57–59]. Our initial analysis in human tumors showed that both genes are negatively correlated in more than 75% percent of the considered datasets (72 out 95 different datasets). In addition, their expression in non-tumoral samples, which is close to 75%, shows that this opposite regulation is a common effect in human cells. Their role as negative regulators of the Notch pathway likely requires a higher transcription of one of the genes than the other, allowing only one of the two proteins to effectively perform its regulatory role. This notion is supported by development studies conducted in mouse and chicken embryos, where NUMB is broadly expressed, while NUMBL appears to be enriched in the developing central nervous system [9–11].

Our previous results have focused on the downregulation of *NUMB* or *NUMBL* by shRNA [17, 48]. We showed that only that the downregulation of only *NUMB* or *NUMBL* causes an increment in the tumorigenic properties of cells, allowing us to focus on their roles as tumor suppressor genes. Therefore, cells with lower NUMBL levels are more resistant to drugs that are commonly used in cancer chemotherapy, such as doxorubicin or vincristine [17]. In fact, loss of NUMBL protein has been associated with an increase in metastasis and worse survival [60].

Our bioinformatics analysis allowed us to obtain an unexpected network of gene correlations with *NUMB* or *NUMBL*. Due to their accepted roles as genes with similar functions, we expected to find common correlations for both genes in different tumors. However, our analysis showed that *NUMB* and *NUMBL* exhibited almost independent correlations when we considered the four tumor types. This result could be due to different regulatory mechanisms, depending on the tissue in which *NUMB* or *NUMBL* is expressed. A comparison of each tumor type allowed us to identify a small number of genes that were equally related to *NUMB* and *NUMBL*, different genes related to similar functions, and genes with differential expression. GO-terms obtained with the different lists of genes that were correlated to *NUMB* described functions such as endocytic protein with important roles in protein transport [2, 3, 44, 45]. GO-terms related to *NUMBL* were related to gene transcription and some of its previously described roles [17, 31, 61]. In addition, common GO and Reactome terms obtained from negative correlations to *NUMB* or *NUMBL* proceeded from different list of genes, pointing to a different gene regulation network. These data may confirm the different cellular roles of each protein.

**Figure 8:**
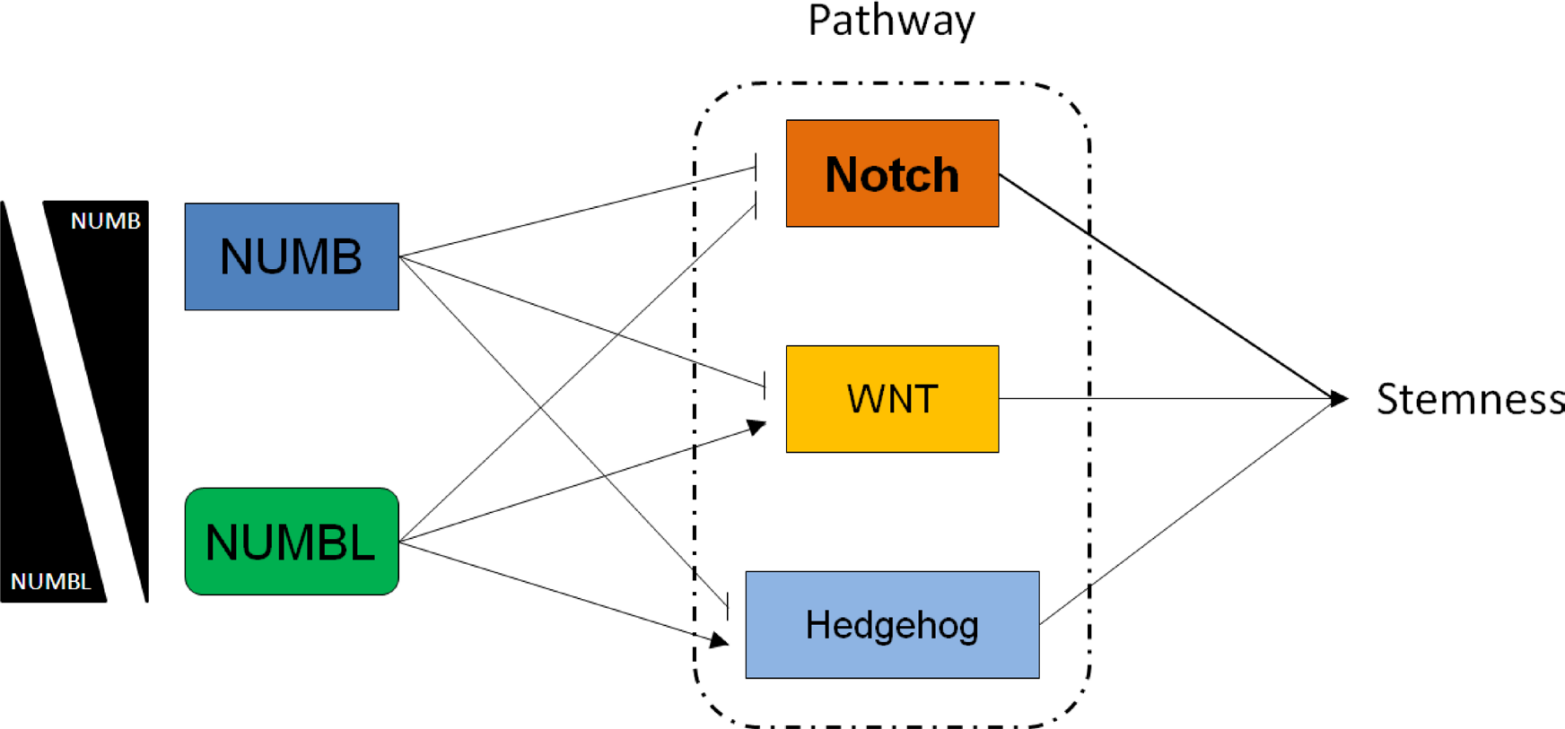
NUMB and NUMBL affect Notch, WNT and Hedgehog pathways differentially

The Notch pathway is involved in the maintenance of tumor stemness and cancer metastasis. This pathway is activated in different tumors, such as lung, colon, breast and prostate tumors, and in sarcomas, melanomas, leukemias and lymphomas [58, 59, 62–65]. In addition, Notch activity has been linked to cancer metastasis by inducing EMT, tumor angiogenesis processes and anoikis resistance of tumor cells [66, 67]. After its interaction with a ligand, Notch is proteolytically cleaved, and NICD is released into the cytoplasm, allowing its nuclear translocation [68, 69]. We have recently showed that NUMBL behaves, such as NUMB, its close relative, as a tumor suppressor gene regulating the Notch pathway. Here, we showed that NUMB and NUMBL presented mutual exclusivity in tumors, suggesting that the tumor suppressor effect on the Notch pathway is regulated by one protein at a time, thus simplifying the regulatory network. In addition, *NUMB* and *NUMBL* also showed opposite correlations in non-tumoral samples, suggesting that this opposite relationship is a common effect in cells. However, the two proteins, which share approximately 55% of their amino acid sequence, did not show total redundancy, as deduced from the differences in mRNA levels observed for genes belonging to the NFκB, ERBB/HER, Hippo, WNT and Hedgehog pathways. Thus, our results obtained by downregulation of *NUMBL* are consistent with previously published studies showing that NUMBL negatively influences the NFκB pathway and positively affects the Hedgehog pathway [31, 32]. In addition, although GLI1 and GLI2 should have similar behaviors, considering their transcription is activated by Hedgehog signaling and appear to be downregulated when the Hedgehog pathway is inactive, GLI1 is also connected to Notch signaling through HES1 inhibition [70, 71]. The large number of genes that are transcriptionally affected in an opposite manner suggests that NUMB and NUMBL have different effects on the signaling pathways (Figure 8). Since the phenotypic effect of *NUMB* and *NUMBL* downregulation is similar, inducing CSC-like properties, this finding suggest a hierarchy of Stem Cell pathways in which Notch pathway activation predominates over the other affected pathways, inducing an increase in stemness due to the lower levels of NUMB or NUMBL.

A possible explanation for the different effects on different pathways, correlating genes to either an oncogene or a tumor suppressor, may be plausible if we consider recent results correlating the alternative splicing of *NUMB* in tumors. p72/p71 NUMB isoforms have been shown to be induced by RAS-ERK signaling in breast and lung cancer [49]. An analysis of all NUMB and NUMBL protein isoforms has shown that only NUMB p72/p66 exhibits the entire sequence of the PTB domain, with the absence of an 11-amino-acid sequence starting after Ala67 in the other two isoforms, p71/p65. In addition, only p72/p71 exhibits the 48-amino-acid sequence that corresponds to exon 9, which has been linked to an increase in tumorigenic properties [49]. NUMBL also lacks this region, although protein homology between NUMB and NUMBL in this region is low, sharing less than 30% of the sequence between Ser373 and Leu574 from NUMBL. Thus, it is plausible that the NUMB and NUMBL isoforms are essential in terms of their different regulatory effects on the properties of cancer.

Expression data from public datasets, although a powerful tool to obtain useful information, may not detect alternative splicing. Thus, although both NUMB and NUMBL are considered tumor suppressors, an increment in the expression of at least *NUMB* cannot be assumed to lead to an increase in a tumor suppressor gene due to the observed alternative splicing. In accordance with this hypothesis, *NUMB* levels are higher in astrocytomas and cervical squamous carcinoma cells, and the positive effect of NUMBL over SHH signaling can also promote tumor progression [23, 33, 34, 49]. Therefore, explorations of the effects of NUMB and NUMBL should not only consider the expression of both genes individually but also the relative levels of their different isoforms.

Alternatively, these differences might be derived from differences in the affinity of NUMB/NUMBL for its targets. In agreement with the previous hypothesis, NICD1 and NICD2, the processed intracellular domains of Notch1 and Notch2, showed similar effects over HES1 induction, while transcription of sequences upon the four tandem CSL binding sites, typical of the promoters of other genes, were different [72]. In addition, the four NICD domains have been shown to be targets for different post-translational modifications, from ubiquitination to acetylation. The latter is particularly important because NICD1 acetylation has been shown to provide protection against degradation [34, 73]. Therefore, it is possible that NUMB and NUMBL preferentially target a different NICD member (not acetylated), explaining the observed differences in gene expression. In addition, Notch receptors have been shown to be differentially affected by Numb in mice [74]. Considering the presence of four different NUMB isoforms, NUMBL, the four Notch receptors and Notch’s multiple post-translational modifications, a very complex scenario can be envisioned that still requires a great deal of research to disentangle.

In summary, these results indicated that NUMB and NUMBL could differentially modulate different signaling pathways in a very different fashion, indicating that, despite the previously observed exclusivity, the full redundancy does not exist between these two genes. Only in the Notch pathway, equal regulation is maintained. However, the similar phenotypic effect is observed under the downregulation of each protein independently, suggesting that Notch pathway regulation may predominate hierarchically in the regulation of the CSC phenotype.

## MATERIALS AND METHODS

### Bioinformatics analysis

To determine the correlation between *NUMB* and *NUMBL* genes in acute amyloid leukemia (AML), acute lymphoblastic leukemia (ALL), chronic lymphocytic leukemia (CLL), ovarian, colon/rectal, lung, renal, breast, neuroblastoma and esophagus tumors, a total of 95 different databases and 19 normal datasets were analyzed using R2 software (Genomics Analysis and Visualization Platform, http://r2.amc.nl). All datasets are freely available at the R2 webpage. To perform these correlations, the 209073_s_at probe was used for *NUMB* and the 242195_x_at for *NUMBL*. In addition, for *NUMB* vs. *NUMBL* heat maps, we used TCGA datasets for Colon Adenocarcinoma and Kidney Renal Clear Cell Carcinomas, Bild dataset for tumor lung (GSE3141), and the EXPO dataset for breast and endometrium tumors (GSE2109).

To analyze the methylation state of *NUMB* and *NUMBL* in human samples, we used the TCGA Wanderer resource (http://maplab.imppc.org/wanderer/) [42], using the datasets for Breast Invasive Carcinoma, Colon Adenocarcinoma, Rectum Adenocarcinoma, Lung Adenocarcinoma, Lung Squamous Cell Carcinoma and Cervical squamous cell carcinoma and endocervical adenocarcinoma. Only CG probes with beta value higher than 0.1 were considered for the analysis.

To identify genes correlated with *NUMB* and *NUMBL*, a total of 28 databases of different tumors (breast, lung, colon and cervix; Supplementary Table 1) were analyzed using R2 software. All datasets are freely available at the R2 webpage. We looked for correlations using probles 209073_s_at (*NUMB*) or 242195_x_at (*NUMBL*) for all Affymetrix datasets. For the Budinska dataset, we used the ADXCRIH.2944.C1_at probe for *NUMB* and the ADXCRAG_AF015041_at probe for *NUMBL*, while for the TCGA datasets, we used the NUMB_8650 probe for *NUMB* and the NUMBL_9253 probe for *NUMBL*. In all cases, we established a *p*-value lower than 0.05 to identify significant differences.

From the list of correlated genes, we separated genes that were positively correlated to NUMB or NUMBL from genes that were negatively correlated to NUMB or NUMBL, generating two gene lists for each database and gene. Next, we searched for genes that were highly represented among the different datasets. Thus, we established a cutoff for each gene of appearing at least in two different databases in cervical cancer, three different databases in lung cancer, four different databases in breast cancer and six different databases in colon cancer. We thereby generated four groups of genes that were commonly negatively or positively correlated to NUMB or NUMBL. To generate a Venn diagram to identify common genes that were correlated to NUMB or NUMBL for all tumors, we used the tool Venny [75].

To identify the pathways or Gene Ontology (GO) terms linked to genes that were positively or negatively correlated to NUMB or NUMBL, we used enrichment analysis from the Gene Ontology consortium webpage (http://geneontology.org/page/go-enrichment-analysis). In addition, we used Reactome (https://reactome.org/) to find altered pathways correlated to NUMB or NUMBL. In both cases, we only take into account genes with a *p*-value lower than 0.05. For Reactome pathways, in addition, we only considered pathways with a FDR lower than 0.05.

To detect correlations between *NUMBL* and genes related to the Notch, WNT or Hedgehog pathways, we used the same previously considered 28 databases of different tumors (breast, lung and colon; Supplementary Table 1), fixing a *p*-value lower than 0.05 to identify statistically significant correlations.

### Cell lines and cellular assays

T47D and HeLa cells were obtained from the European Collection of Authenticated Cell Cultures (ECACC) commercial repository at the beginning of this study. No further authentication was performed for these cell lines. Cells were maintained in DMEM (Sigma) supplemented with 10% FBS (Life Technologies), penicillin, streptomycin, and fungizone. To downregulate *NUMB* and *NUMBL* expression, we used shRNA plasmids from Origene (TR311064, TR311063, Rockville, MD) as previously described [17, 48]. All transfected cells were selected with 1 μg mL^−1^ of puromycin.

### Protein isolation and western blot analysis

Protein extracts for Western blot analysis were obtained as described previously [17]. For Western blot detection, we used NUMB (ab4147, Abcam, 1 μg/mL) and NUMBL (ab37500, Abcam, 1 μg/mL) antibodies. α-Tubulin (T9026, Sigma) was used as a control. Horseradish peroxidase-labeled rabbit anti-mouse (ab97046, Abcam, diluted 1:5,000) and goat anti-rabbit (ab97051, Abcam, diluted 1:5,000) secondary antibodies were used.

### Analysis of gene transcription

Total RNA was purified as described previously [17]. To detect changes in gene expression, we used the following probes, all from Life Technologies: *NUMB* (Hs01105433_m1), *NUMBL* (Hs00191080_m1), *HES1* (Hs00172878_m1), *HES5* (Hs01387463_g1), *KLF7* (Hs00748636_s1), *NFKB1* (Hs00765730_m1), *ERBB2* (Hs01001580_m1), *TEAD1* (Hs00173359_m1), *TCF4* (Hs00162613_m1), *EN1* (Hs00154977_m1), *BTRC* (Hs00182707_m1), *SOX9* (Hs01001343_g1), *PTCH1* (Hs00181117_m1), *GLI1* (Hs01110766_m1), *GLI2* (Hs01119974_m1), *CDH1* (Hs01023895_m1), *SOX2* (Hs01053049_s1), *BMI1* (Hs00995536_m1), *NANOG* (Hs04260366_g1) and *GAPDH* (Hs03929097_g1). Quantitative PCR was performed as described previously [17].

## SUPPLEMENTARY MATERIALS FIGURES AND TABLES














