# Supplementary material for: NUMB and NUMBL differences in gene regulation
Source: Oncotarget. 2018 Jan 11;9(10):9219–34. doi: 10.18632/oncotarget.24186 (PMC5823667; doi:10.18632/oncotarget.24186)
Supplement: Supplementary file 1 [file oncotarget-09-9219-s001.pdf]

NUMB and NUMBL differences in gene regulation

SUPPLEMENTARY MATERIALS

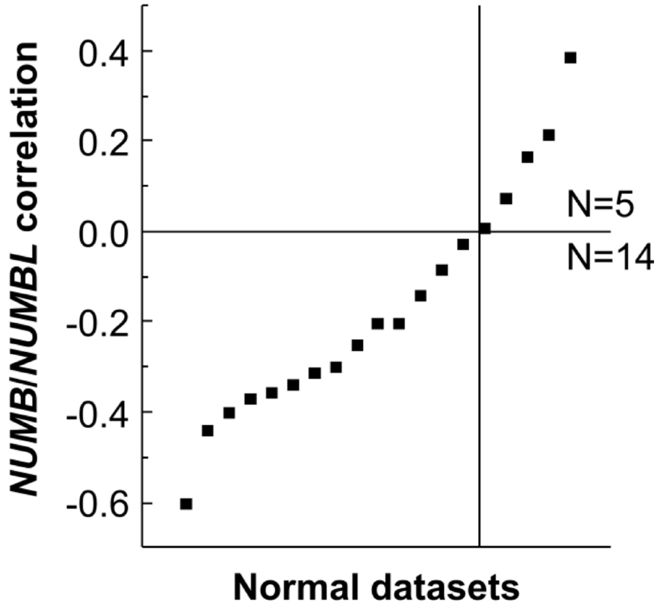

Supplementary Figure 1: *NUMB* and *NUMBL* are negatively correlated in human non-tumoral samples. NUMB/NUMBL correlations in non-tumoral samples, showing a higher percentage of negative correlations.

Supplementary Table 1: List of datasets used in this work

Lung	Cervix	Breast	Colon
GSE43580 (Peitsch)	GSE6791 (Ahlquist)	GSE36771 (Black)	GSE8671 (Marra)
GSE3141 (Bild)	TCGA/CESC/305 samples	GSE12276 (Bos)	GSE21510 (Sugihara)
GSE2109 (EXPO)		GSE16391 (Desmedt)	GSE4554 (Watanabe)
GSE33532 (Muley)		GSE2109 (EXPO)	GSE41568 (Clary)
TCGA/LUAD/515 samples		GSE5460 (Iglehart)	E-MTAB-990 (Budinska)
TCGA/LUSC/81 samples		GSE9195 (Loi)	GSE2109 (EXPO)
		GSE5462 (Miller)	GSE37892 (Olschwang)
		GSE30682 (Servant)	GSE14333 (Sieber)
			GSE17538 (Smith)
			TCGA/COAD/286 samples
			GSE39582 (Marisa)
			GSE13294 (Jorissen)

Supplementary Table 2: List of genes correlated to *NUMB* or *NUMBL*. See Supplementary_Table_2

Supplementary Table 3: Number of genes correlated with *NUMB* or *NUMBL* in individual tumor type

	Colon		Lung		Breast		Cervix	
	NUMB+	*NUMB-*	*NUMB+*	*NUMB-*	*NUMB+*	*NUMB-*	*NUMB+*	*NUMB-*
NUMBL+	228	135	316	184	90	34	25	8
NUMBL-	415	232	248	108	98	113	11	16

+: positive correlation; -: negative correlation.

Supplementary Table 4: GO and Reactome Pathway terms obtained from genes positively correlated with *NUMBL*. See Supplementary_Table_4

Supplementary Table 5: GO and Reactome Pathway terms obtained from genes negatively correlated with *NUMBL*. See Supplementary_Table_5

Supplementary Table 6: GO and Reactome Pathway terms obtained from genes positively correlated with *NUMB*. See Supplementary_Table_6

Supplementary Table 7: GO and Reactome Pathway terms obtained from genes negatively correlated with *NUMB*. See Supplementary_Table_7

Supplementary Table 8: List of common GO and Reactome terms obtained from genes negatively correlated both with *NUMB* and *NUMBL*. See Supplementary_Table_8
